# The Placenta as a Target of Epigenetic Alterations in Women with Gestational Diabetes Mellitus and Potential Implications for the Offspring

**DOI:** 10.3390/epigenomes5020013

**Published:** 2021-05-10

**Authors:** Dennise Lizárraga, Alejandra García-Gasca

**Affiliations:** Laboratory of Molecular and Cellular Biology, Centro de Investigación en Alimentación y Desarrollo, Mazatlán, Sinaloa 82112, Mexico; elda.lizarraga.dc19@estudiantes.ciad.mx

**Keywords:** epigenetic inheritance, placental alterations, gestational diabetes mellitus, placental epigenetic alterations

## Abstract

Gestational diabetes mellitus (GDM) is a pregnancy complication first detected in the second or third trimester in women that did not show evident glucose intolerance or diabetes before gestation. In 2019, the International Diabetes Federation reported that 15.8% of live births were affected by hyperglycemia during pregnancy, of which 83.6% were due to gestational diabetes mellitus, 8.5% were due to diabetes first detected in pregnancy, and 7.9% were due to diabetes detected before pregnancy. GDM increases the susceptibility to developing chronic diseases for both the mother and the baby later in life. Under GDM conditions, the intrauterine environment becomes hyperglycemic, while also showing high concentrations of fatty acids and proinflammatory cytokines, producing morphological, structural, and molecular modifications in the placenta, affecting its function; these alterations may predispose the baby to disease in adult life. Molecular alterations include epigenetic mechanisms such as DNA and RNA methylation, chromatin remodeling, histone modifications, and expression of noncoding RNAs (ncRNAs). The placenta is a unique organ that originates only in pregnancy, and its main function is communication between the mother and the fetus, ensuring healthy development. Thus, this review provides up-to-date information regarding two of the best-documented (epigenetic) mechanisms (DNA methylation and miRNA expression) altered in the human placenta under GDM conditions, as well as potential implications for the offspring.

## 1. Introduction

Gestational diabetes mellitus (GDM) is a worldwide health concern affecting pregnant women and their offspring. In 2019, this pregnancy complication affected 17 million newborns globally [1]. The Diabetes Atlas published in 2019 by the International Diabetes Federation [1] reported that 15.8% of live births in 2019 were affected by hyperglycemia during pregnancy, of which 83.6% were due to gestational diabetes mellitus, 8.5% were due to diabetes first detected in pregnancy, and 7.9% were due to diabetes detected before pregnancy, increasing the susceptibility to developing chronic diseases in their adult life [2]. Obesity, malnutrition, excess gestational weight gain, sedentary lifestyle, and stress favor insulin resistance in pregnancy and the development of GDM. GDM may disappear after labor, but women that presented GDM while pregnant may have a higher risk of developing type II diabetes, preeclampsia, and preterm birth [3].

Under GDM conditions, the placenta suffers morphological and structural modifications affecting its function [4,5]. High concentrations of different molecules and nutrients such as glucose, free fatty acids (FFA), insulin, and proinflammatory cytokines cause placental dysfunction, which may affect the fetus [6]. Some of these alterations affect labor outcome as in stillbirth [7] and dystocia [8]. The newborn affectations include macrosomia, hyperinsulinemia, and hypoglycemia [6]. They also become susceptible to developing cardiovascular diseases [9], obesity [10], type II diabetes [11], and metabolic disorders [12] in adulthood. This is consistent with the fetal origins of adult disease (FOAD) hypothesis, first described by Barker and coworkers in the 2000s [13,14].

The mechanisms via which the fetus is programmed to disease in adult life are not well understood. Some investigations suggest that this programming occurs due to inherited epigenetic marks [15] and microbiota seeding [16]. Epigenetics is the study of stable changes in gene function without DNA sequence modifications. Some theories have been proposed to explain how an individual could acquire these epigenetic marks, including (1) direct exposure to the environment throughout life, (2) events occurring during gestation affecting the fetus, and (3) transgenerational inheritance from the parental germline [17]. These modifications are not permanent but could persist for generations [18] as observed in plants and animal models. Whether transgenerational inheritance of epigenetic marks occurs in humans, and how it differentiates from the impact of the environment and the lifestyle remain unclear and require further investigation [19].

The placenta favors epigenetic flow during gestation [20], controlling both the pregnant woman’s metabolism and fetal development. We define this epigenetic flow as all epigenetic alterations induced by the mother’s condition, reaching the fetus through the placenta. Alterations in this epigenetic flow may induce pregnancy complications and disease susceptibility for both the mother and the baby [21]. GDM can trigger epigenetic alterations in the placenta [22], such as DNA methylation variations [23], and misexpression of noncoding RNAs, of which the best studied are miRNAs [24]; these epigenetic mechanisms modulate gene expression and placental function.

## 2. Insulin Sensitivity in Pregnancy

Insulin sensitivity is related to the cellular response to insulin [25]. This process is adjusted during pregnancy to support fetal and placental development. In early gestation, both hypertrophy and hyperplasia of pancreatic β-cells occur, increasing insulin levels by around 60% to equilibrate glucose intake [26]. At the end of the second trimester, insulin sensitivity begins to decrease, and, during the third trimester, insulin sensitivity lowers to 45–70%, relative to nonpregnant women [27] in liver, muscle, and adipose cells. As a result, the liver increases gluconeogenesis, while the muscle stops producing glycogen, increasing glucose concentrations in the bloodstream. At the same time, the adipose tissue stops storing lipids and sets off lipolysis, increasing the levels of FFA [28]. Maternal insulin resistance (IR) ensures the utilization of fats rather than carbohydrates to provide the mother with energy and, at the same time, make carbohydrates available to the fetus. This feature during pregnancy ensures a ready supply of energy to the fetus [29].

### The Role of the Placenta

**Figure 1:**
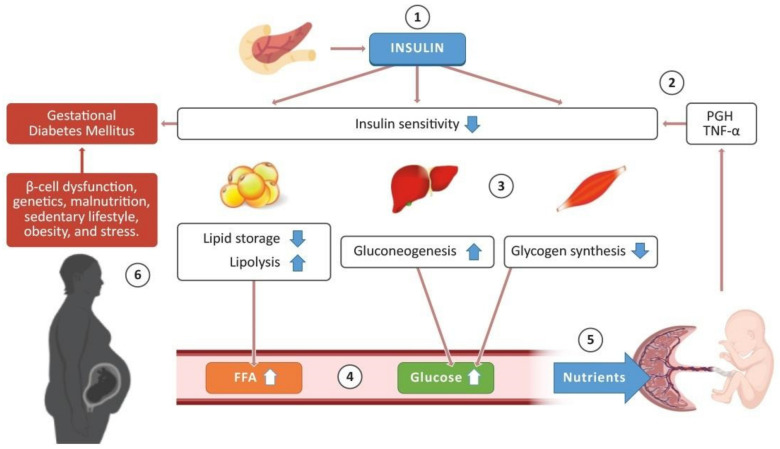
Schematic representation of the glucose-insulin metabolism in pregnancy. The pancreas releases insulin, which promotes different processes in some tissues to regulate glucose concentration in the bloodstream (1). During a healthy pregnancy, the placenta produces the placental growth hormone (PGH) and proinflammatory cytokines such as tumor necrosis factor-alpha (TNF-α), promoting a decrease in insulin sensitivity in adipose tissue, liver, and skeletal muscle (2). Consequently, adipose tissue stops lipid storage and activates lipolysis; the liver enhances the production of endogenous glucose (gluconeogenesis), and glycogen synthesis decreases in skeletal muscle (3). These processes promote an increase in free fatty acids (FFA) and glucose concentration in the bloodstream (4), which are required as nutrients for the development of the placenta and the fetus (5). Nevertheless, some pregnant women present risk factors that trigger the development of gestational diabetes mellitus (6). Some images in this figure were taken from https://biorender.com/ (last accessed on 25 March 2021).

The placenta is involved in the reduction in insulin sensitivity during pregnancy. Placental growth hormone (PGH) and proinflammatory cytokines play a crucial role in this process. PGH is the product of the *GH-V* gene produced by the placenta, and it differs from the human growth hormone (HGH) produced by the pituitary gland in just 13 amino acids [30]. Barbour et al. [31] observed that PGH stimulates insulin resistance in mice overexpressing the human PGH, which occurs due to the overexpression of the p85α regulatory subunit of the PI3-kinase in the skeletal muscle. The increase in p85α affects p85–p110 heterodimer binding to the insulin receptor substrate (IRS-1) protein and the translocation of the glucose transporter type 4 (GLUT-4) to internalize glucose into the cell [32]. At the same time, proinflammatory cytokines are known to reduce insulin sensitivity during pregnancy. Tumor necrosis factor-α (TNF-α) and cortisol increase during late gestation, while insulin sensitivity decreases [28]. The placenta produces TNF-α, and its concentration in maternal blood is inversely proportional to insulin sensitivity [33]. When TNF-α and other cytokines such as interleukin-6 (IL-6) and interleukin-1β (IL-1β) bind to their receptors, protein kinase C (PKC) is activated, inhibiting IRS-1 [34]. These processes contribute to the decrease of insulin sensitivity during pregnancy, and they provide nutrients such as FFA and glucose to the fetus. Some factors, e.g., β-cell dysfunction, genetics, malnutrition, sedentary lifestyle, obesity, and stress, can trigger insulin resistance and promote GDM [3] (Figure 1). 

## 3. Gestational Diabetes Mellitus and Adverse Effects on the Offspring

The American Diabetes Association [35] defines gestational diabetes mellitus as diabetes diagnosed in the second or third trimester of pregnancy in women who were not diabetic prior to pregnancy. Prenatal control is essential to prevent diabetes during gestation; nevertheless, some pregnant women do not have access to medical services for detecting diabetes on time, or the criteria used to diagnose the pathology are not appropriate. According to the World Health Organization [36], the Association of Diabetes and Pregnancy Study Group (IADPSG) criterion should be used, which consists of an oral glucose tolerance test (OGTT) between 24 and 28 weeks of gestation.

The consequences of GDM affect both the mother and the newborn. The mother has a higher risk of developing preeclampsia and preterm birth during pregnancy [3], as well as type II diabetes later in life [37], while the consequences for the offspring (in the short and long term) are more varied than those for the mother. A retrospective study demonstrated that fetal death rates were higher in diabetic pregnant women than in nondiabetic women; this was particularly evident at approximately 32 weeks of gestation [38]. However, one limitation of this study is that it did not distinguish pregestational diabetes from gestational diabetes. The prevalence of macrosomia in GDM varies according to the region, e.g., 16.4% in patients from USA [39] and 12.9% in patients from Argentina [40]. Macrosomia is a risk factor in shoulder dystocia, which requires additional obstetrical strategies. It may result in nerve palsies of the upper extremities, hypoxic neonatal injury, or neonatal death [8]. The offspring also presents a high risk of hypoglycemia [41] due to the increase in insulin secretion caused by maternal hyperglycemia [42]. 

**Figure 2:**
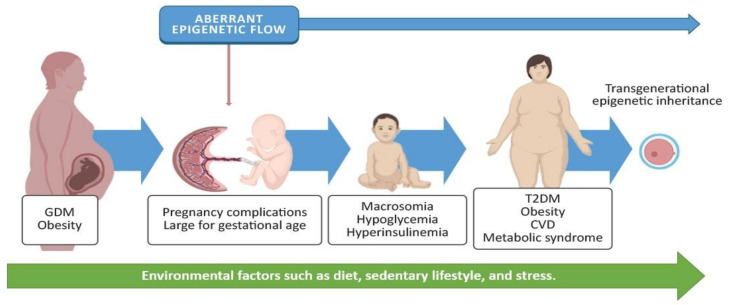
GDM and obesity are chronic diseases often present in pregnant women. These may induce an aberrant epigenetic flow, triggering pregnancy complications and postnatal metabolic disorders for the baby, such as macrosomia, hypoglycemia, and hyperinsulinemia. Environmental factors (such as diet, activity, or stress) may increase the incidence of type 2 diabetes mellitus (T2DM), obesity, cardiovascular diseases (CVD), and metabolic syndrome; they may also promote the inheritance of aberrant epigenetic marks. Some images in this figure were taken from https://biorender.com/ (last accessed on 25 March 2021).

A population-based cohort study with 40 years of follow-up involving offspring exposed to maternal diabetes demonstrated a 29% higher risk of early-onset cardiovascular disease compared with offspring from nondiabetic mothers. Specifically, in the gestational diabetes group, the incidence of cardiovascular disease in the offspring was 1.60 per 1000 person-years [9]. GDM is also related to higher offspring adiposity and greater body mass index (BMI) at the age of 18 [10]. A longitudinal study in the Pima Indian community in Arizona showed that intrauterine exposure to maternal hyperglycemia increased type II diabetes by 40% in 5–19 year old children [11]. These affectations contribute to the development of metabolic syndrome, which Moore [12] defines as “a combination of glucose intolerance, hyperinsulinemia, hyperlipidemia, central obesity, and hypertension”. Recent studies have associated maternal diabetes with the risk of autism spectrum disorders [43] and hypothalamic alterations [44]. These findings demonstrate that exposure to maternal hyperglycemia such as GDM has consequences for the mother during pregnancy and through labor, as well as for the offspring’s lifetime health (Figure 2).

## 4. Alterations in the Placenta in Women with GDM

The placenta is a unique organ that originates only in pregnancy. Its principal role is communication between the mother and the fetus, allowing the exchange of nutrients, gases, and other biomolecules that ensure a healthy development for the fetus and wellbeing for the mother [45]. Hence, any disturbance to the placenta could affect both the fetus and the mother.

As a consequence of GDM, the placenta may undergo different alterations [46,47]. Morphologically, the placenta presents a variety of abnormalities. Daskalakis et al. [48] recruited 80 singleton pregnancies (40 with GDM and 40 normal), with uncomplicated and in-term deliveries. GDM placentas showed (1) higher placental weights, (2) presence of degenerative lesions like villous fibrinoid necrosis, (3) vascular lesions such as chorangiosis, (4) villous immaturity, and (5) chronic fetal hypoxia according to the presence of nucleated fetal red blood cells. The higher placental weight in GDM was probably due to a decrease in the apoptotic activity, where the mitochondrial antiapoptotic B-cell lymphoma-2 (BCL2) protein increased and caspase-3 decreased [49], indicating intrinsic apoptosis inactivation via BCL2. The placenta also produces proinflammatory cytokines during GDM. It has been shown that TNF-α levels are higher in placentas from women with GDM compared to women with normal glycaemia; 94% of TNF-α production is derived from the maternal side of the placenta, and its concentration in the mother’s blood is proportional to insulin resistance [33], indicating that placental production of TNF-α promotes insulin resistance in the mother.

The placenta ensures fetal survival by adapting to intrinsic (gestational age, genetic, etc.) or extrinsic (environmental, nutritional, stress, etc.) factors. These adaptations depend on the severity and time of exposure [50]. In GDM cases, alterations in the placenta might be interpreted as adaptations to protect the fetus from an aberrant environment, impacting nutrient and molecule transport [51], which may result in neonate complications and diseases later in life.

The placental plasticity to modify mechanisms that can program the fetus to be prone to diseases later in life is poorly understood. Some investigations suggest that this programming occurs due to epigenetic changes during gestation and early postnatal life [15]. In this context, the “three-hit” hypothesis explains the predisposition to certain phenotypes due to the (sequential) interaction of genetic and environmental factors [52]. Briefly, this hypothesis mentions that the genetic predisposition (hit-1) interacts with the early-life environment (hit-2, which may include epigenetic modifications during gestation), programming the fetus to a certain phenotype, followed by exposure to environmental factors later in life (hit-3). Thus, under GDM conditions, the fetus has already been subjected to hit-2, and the phenotype will vary depending on the environmental factors to which the individual is exposed throughout life (hit-3).

### 4.1. Epigenetic Modifications in the Placenta Caused by GDM

Epigenetics is the study of stable changes in gene function without DNA sequence modifications. These stable changes include several mechanisms such as DNA and RNA methylation, chromatin remodeling, histone modifications, and the expression of noncoding RNAs (ncRNAs). The placenta presents its own epigenetic programing during gestation [20], affecting both the pregnant woman’s metabolism and fetal development. Thus, epigenetic alterations will likely induce pregnancy complications and disease susceptibility for both [21] (Figure 2). As a consequence of GDM, the placenta undergoes epigenetic alterations [22]; most studies regarding these epigenetic alterations focused on DNA methylation [23] and miRNA expression [24], which modulate gene expression and placental function.

### 4.2. Placental DNA Methylation

DNA methylation (DNAm) occurs when a methyl group is covalently transferred to the fifth carbon of a cytosine base (5-methylcytosine or 5-mC). Methylation is achieved by DNA methyltransferase enzymes (DNMTs), and it occurs at different sites within the genome. Commonly, it comes about in the cytosine next to a guanine base in gene promoters, also called CpG islands, regulating gene expression [53]. DNAm occurring in regulatory sequences is associated with chromatin structure; hypermethylation of regulatory elements causes gene silencing through blocking transcription factor-binding sites or through recruiting proteins with a methyl-CpG binding domain (MBD) and histone modification enzymes, causing a tightly packed chromatin conformation (heterochromatin); however, when the regulatory elements are hypomethylated, the chromatin relaxes (euchromatin), enabling gene expression [54]. 

Metabolic genes are conspicuous targets to study epigenetic alterations in placenta due to GDM. During pregnancy, the placenta secretes several molecules, such as leptin, an adipokine involved in metabolic homeostasis, which also regulates placentation and nutrient transport to the fetus [55]. Lesseur et al. [56] measured DNAm at 23 CpGs in the leptin gene (*LEP*) promoter and observed higher methylation in placentas from women with GDM (*n* = 47) compared to placentas from women without GDM, which means a downregulation of the *LEP* gene under GDM conditions. Leptin regulates energy balance suppressing food intake, meaning that a decrease in *LEP* expression would favor obesity [57]; however, the authors [56] did not evaluate expression of the *LEP* gene, which is important to properly interpret the results, since gene expression might be regulated by different epigenetic mechanisms. Hence, there is still controversy on placental leptin production, since other authors observed that the expression of leptin and its receptor increased in placentas of women with GDM compared with placentas of women without GDM [58]. Despite the controversy, placental leptin plays an important role in modulating implantation, placentation, and cell proliferation, as well as the mobilization of maternal fat [59]. Another important metabolic gene altered during GDM is lipoprotein lipase (*LPL*); DNAm levels were lower in the promoter of the *LPL* gene in placentas from women with GDM, which was negatively correlated to the mRNA levels in the placenta. Moreover, DNAm patterns positively correlated to anthropometric characteristics in 5 year old children exposed to GDM, such as birth weight, mid-childhood weight, and fat mass [60]. LPL is an extracellular enzyme whose key function is triglyceride hydrolysis into fatty acids in the bloodstream [61]; thus, increased *LPL* expression levels in the placenta may indicate a major supply of fatty acids to the fetus. These findings indicate that GDM alters DNAm in the promoters of metabolic genes, which may predispose the fetus to metabolic diseases later in life. 

One study analyzed DNAm in different DNA regions [62]; the authors collected cord blood and chorionic villus tissue from 88 women dietetically treated for GDM, 98 women with insulin-dependent GDM, and 65 women without GDM. Results showed a significant decrease in methylation levels in three important DNA regions in both GDM groups: (1) the maternally imprinted *MEST*, (2) the glucocorticoid receptor *NR3C1*, and (3) *ALU* repeats. Hypomethylation of *MEST* was also found in the blood of obese adults, which has been linked to body weight increase in mice [63]. El Hajj et al. [62] suggested that *NR3C1* hypomethylation could lead to an increased exchange of glucocorticoids across the placenta. Alterations in *NR3CI* methylation have been associated with infant neurobehavior [64] and with prenatal exposure to maternal depression [65]. Lastly, lower DNA methylation in *ALU* repeats may indicate genome-wide alterations under GDM conditions. *ALU* repeats are transposable elements, and hypomethylation is associated with epigenome instability and disease [66]. 

**Figure 3:**
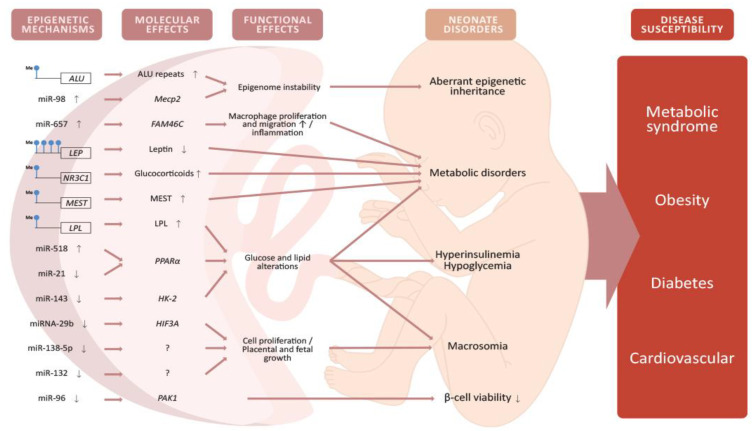
Alterations of DNA methylation and miRNA expression in the placenta from women with GDM will alter the expression and function of genes involved in metabolic and cellular pathways. These alterations will have potential implications for the offspring such as hyperinsulinemia, hypoglycemia, macrosomia, and lower viability of pancreatic β-cells. These neonate disorders may, in turn, produce an increased susceptibility to developing obesity, diabetes, metabolic syndrome, and cardiovascular diseases later in life. Some images in this figure were taken from https://biorender.com/ (last accessed on 25 March 2021).

Global DNA methylation refers to the presence of 5-mC in the whole genome [67]. To date, two investigations exist regarding global DNA methylation in the placenta from GDM with contradictory results. In a pilot study, [68] examined 50 placentas, eight from women with GDM. Global DNA methylation was evaluated using a semiquantitative luminometric methylation assay (LUMA), detecting lower global DNA methylation in placentas with GDM, but higher methylation in umbilical cord blood. A more recent study [69] analyzed global DNA methylation through LC–ESI/MS/MS (liquid chromatography–electrospray ionization/multistage mass spectrometry) in 1030 placentas, of which 56 were from women with GDM, and it demonstrated that global DNA methylation in the placenta was higher under GDM conditions compared to non-GDM. These contradictory results may be due to differences in sample size and the methods used. Despite the contradiction, these studies indicate that the placenta undergoes epigenetic reprogramming under GDM conditions, which may show a relationship with newborn metabolic and anthropometric alterations (Figure 3). Interestingly, a placental DNA methylation signature has been linked to maternal insulin sensitivity through a comprehensive DNA methylation array covering more than 720,000 CpGs across the genome [70], which could be relevant in terms of GDM. More studies with standardized methodologies and a representative sample size are needed to provide more consistent information and to determine whether global DNA methylation changes are caused by GDM in the placenta. A summary of documented DNA methylation changes in the placenta under GDM conditions is presented in Supplementary Table S1 (Placental DNA methylation).

### 4.3. miRNA Expression in the Placenta

Noncoding RNAs (ncRNAs) are functional RNA molecules that are transcribed but do not encode a protein. While epigenetic mechanisms involve modifications in DNA and RNA (methylation) and/or histones (methylation, acetylation or other post-translational modifications) to regulate gene expression, ncRNAs are involved in gene regulation at the transcriptional and post-transcriptional levels; they may not be considered epigenetic regulators per se, but they do present altered expression profiles in GDM (Supplementary Table S1, Placental miRNA expression) which could be related to changes in DNA methylation. ncRNAs include microRNAs (miRNAs) which regulate gene expression in a specific manner [18]. There is evidence that shows the important role of miRNAs in pregnancy homeostasis [71], and that dysregulation could be related to GDM [24]. It has been reported that miRNAs can indirectly modulate global DNA methylation; for instance, expression of miR-98 in placental tissue from GDM women was related to an increase in global DNA methylation by targeting the methyl-CpG binding protein 2 (*MECP2*) gene [72]. MECP2 is a protein with a methyl-CpG binding domain that binds to regulatory elements blocking transcription factor binding, which favors heterochromatin formation [73]. Alterations in the *MECP2* gene may result in neurodevelopmental disorders such as Rett Syndrome [74]. 

Genes involved in lipid and glucose metabolism are also targets of placental miRNAs. Zhao et al. [75] investigated the expression of miR-518d in placentas from 40 women with GDM and C-section delivery, compared to 40 normal glucose tolerance (NGT) patients. The authors reported upregulation of miR-518d, which negatively regulates the peroxisome proliferator-activated receptor-α (*PPARα*) gene in the placenta of patients with GDM. PPARα is a nuclear receptor involved in lipid, glucose, and inflammation homeostasis, and it plays an important role in diabetic cardiomyopathy homeostasis [76]. Nevertheless, a recent study with 137 patients with GDM and 158 NGT women reported downregulation of miR-21 and an increase in PPARα in placentas derived from women with GDM compared to the control group [77]. On the other hand, miR-143 was downregulated in women with GDM treated with insulin, compared to women treated with a diet and to NGT patients; miR-143 targets the hexokinase-2 (*HK-2*) enzyme [78], which phosphorylates glucose to produce glucose-6-phosphate, the first step in the glycolytic pathway [79]. Another study using HTR-8/SVneo and BeWo cells reported that high-glucose treatment suppressed cell viability and reduced miR-132 expression, concluding that miR-132 may enhance trophoblast proliferation, playing a protective role against GDM [80]. Thus, placental miRNAs are involved in lipid and glucose metabolism, and misexpression may contribute to GDM pathogenesis and adverse offspring outcomes (Figure 3).

Placental miRNAs may protect pancreatic β-cells in women with GDM. Li et al. [81] evaluated miR-96 expression in placentas from three women with GDM and three NTG patients. miR-96 was downregulated in the GDM group and was negatively correlated with blood glucose concentration. The target gene of this miRNA is p21-activated kinase 1 (*PAK1*), which showed increased expression levels in plasma from the GDM group. PAK1 is a serine/threonine kinase important in the proliferation and function of β-cells in mice and humans [82]. Moreover, a functional assay with INS-1 cells transfected with miR-96 inhibitor showed an inhibition of cell apoptosis and improved viability, whereas cells transfected with si-PAK1 showed the opposite effect. It has also been shown that cell proliferation and infiltration are altered by miRNAs in placentas from women with GDM. miRNA-29b was downregulated in placental tissue from GDM patients compared to healthy placentas, while increasing the expression of its target gene, the hypoxia-inducible factor 3 subunit alpha (*HIF3A*), showed that the inhibition of miRNA-29b in the trophoblast HTR8/SVneo cell line enhanced cell proliferation and infiltration [83]. In addition, the mRNA levels of *HIF3A* increased in subcutaneous adipose tissue of obese people and promoted adipose tissue dysfunction [84]. Similarly, the expression of miR-657 in mononuclear macrophages isolated from placental tissue was higher in the GDM group compared to the NGT group, targeting the noncanonical poly(A) polymerase *FAM46C*, which resulted in proliferation, migration, and polarization of the macrophages [85]. FAM46C is a poly(A) polymerase that acts as an onco-suppressor in B-lymphocyte lineages, and loss of function drives myeloma development [86]. Therefore, variations of placental miRNA expression under GDM conditions may alter the behavior of different cell types. 

Technologies for the analysis of gene expression profiles (such as miRNA microarrays and miRNA-seq) have been used to identify miRNAs expressed in GDM placental tissue. Li et al. [87] studied the expression of miRNAs in 15 placentas from women with GDM and 15 from NGT patients. The analysis identified differential miRNA expression in GDM placental tissue, where one miRNA was upregulated and eight were downregulated. The authors reported that the target genes of these miRNAs were involved in the epidermal growth factor receptor (EGFR)/PI3K/Akt pathway in placental and fetal development, and they presented a model proposing that enhanced EGFR signaling exists in tissues under GDM conditions, contributing to enhanced fetal and placental growth, resulting in macrosomia, since activation of the PI3K/Akt pathway enhances cell proliferation, survival, and growth (among other functions) [88]. Similar findings were observed in chorionic villi explants from 12 women with GDM and 12 healthy placentas using miRNA-seq [89]. The authors observed a different miRNA exosome profile in the GDM group, with nine upregulated and 14 downregulated miRNAs, targeting signaling pathways associated with PI3K/Akt and mitogen-activated protein kinase (MAPK)/ERK1/2, both involved in cell proliferation [88,90]. They concluded that placental exosomes might play a role in the modulation of insulin sensitivity in normal and GDM pregnancies. Another study from Ding et al. [91] evaluated the miRNA profiles of HTR-8 cells derived from chorionic villi explants from human first-trimester placentas (eight women with GDM and eight healthy pregnancies). Despite the large profile of miRNAs found in the analysis, they focused on miR-138-5p, which showed a decreased expression in GDM HTR-8 cells compared to the control cells, resulting in higher proliferation and migration of trophoblasts. More recently, a study examined the miRNA profiles in the placental tissue from 57 GDM and 61 NGT pregnant women [92]. Results were further confirmed with qRT-PCR, which showed upregulation of miRNA-144 and downregulation of miRNA-125b in placentas from women with GDM compared with the control group. The expression of those miRNAs was similar to circulating exosomes in plasma from women with GDM. The upregulation of miRNA-144 was negatively correlated to the body mass index (BMI) and positively linked with blood glucose tolerance at 1 h in the OGTT. All these results indicate that placental miRNAs play an important role in GDM pathogenesis, by modulating metabolic genes and cellular pathways in pregnant women, involved in the function and behavior of pancreatic β-cells. 

Investigations regarding ncRNAs (other than miRNAs) in the placenta under GDM conditions are scarce. The whole transcriptome of the maternal surface of the placenta was recently analyzed by high-throughput sequencing [93]. The work showed that the placentas from women with GDM presented 290 differentially expressed ncRNAs compared to the control group. Upregulated ncRNAs under GDM conditions were composed of two miRNAs, 86 long noncoding RNAs (lncRNAs), and 55 circular RNAs (circRNAs), while downregulated ncRNAs included another two miRNAs, 86 lncRNAs, and 59 circRNAs. This reveals a differential transcriptomic profile in the placenta under GDM conditions compared with normoglycemic conditions. circRNA expression profiles of GDM have also been sequenced in the placenta. Yan et al. [94] identified a total of 482 circRNAs differentially expressed in the placental villi of GDM women, of which 227 were upregulated and 255 were downregulated. With the aid of Gene Ontology and the Kyoto Encyclopedia of Genes and Genomes, the authors determined that these circRNAs were related to glucose and lipid metabolism. The characterization of histone modifications and emerging epigenomic research, including RNA methylation [95] and single-cell RNA sequencing [96] in the placenta, are needed to further understand GDM and its impacts on the offspring.

### 4.4. Epigenetic Alterations in the Offspring and Potential Implications 

Little is known about the epigenetic signature acquired by the offspring exposed in utero to GDM conditions. Epigenome-wide association studies (EWAS) have been used to understand the basis for disease risk in the epigenetic epidemiology field. Howe et al. [97] carried out a meta-analysis from EWAS of seven cohort participants in the Pregnancy and Childhood Epigenetics (PACE) consortium focusing on the study of the umbilical cord blood. The seven cohorts included 3677 participants from eight countries, of which 317 were in the GDM group and 3360 were in the control group. The meta-analysis was adjusted to multiple covariates and identified two differentially methylated regions in the GDM group compared to the control group: (1) lower methylation in the promoter of *OR2L13*, an olfactory receptor gene associated with autism spectrum disorder, and (2) lower methylation within the cytochrome P450 (*CYP2E1*) gene, which encodes a metabolic enzyme in the liver with increased expression in diabetic patients. Kim et al. [98] performed EWAS to evaluate the DNA methylation profile of children exposed in utero to maternal GDM. The participants were sibling pairs, 18 exposed to GDM in utero (aged 5.8 ± 1.4) and 18 not exposed (aged 9.4 ± 2.5). The genomic DNA was extracted from peripheral blood leukocytes, and the methylation profile was measured using the Infinitum HumanMethylation450 BeadChip (HM450K array). They identified 12 differentially methylated CpG sites in the GDM-exposed group, two of which have been linked to monogenic diabetes (hepatocyte nuclear factor 4 alpha, *HNF4A*) and obesity (Ras responsive element binding protein 1, *RREB1*). A more recent study [99], also applying the HM450K array, evaluated the DNA methylation profile in peripheral blood of 93 offspring exposed to GDM and 95 not exposed, aged from 9 to 16 years, from the Danish National Birth Cohort. The authors found 76 differentially methylated CpG sites in the offspring exposed to GDM, of which nine (the 11.8%) were within genes involved in type 2 diabetes, obesity, diabetic nephropathy, or coronary heart disease. On the other hand, miRNAs involved in insulin sensitivity were misexpressed in the offspring exposed to maternal GDM. Houshmand-Oeregaard et al. [100] studied the second follow-up of a cohort of 206 adult offspring (aged between 26 and 35 years) born to women with diabetes during pregnancy, of which 82 were offspring of women with gestational diabetes (O-GDM), 62 were offspring of women with type 1 diabetes (O-T1DM), and 57 were from the control group. The expression of miR-15a and miR-15b increased in skeletal muscle biopsies of the participants in the O-GDM and O-T1DM groups compared with the control group, which was positively linked with fasting plasma glucose, 2 h plasma glucose, and HbA1c values. These studies suggest that in utero exposure to GDM modulates DNA methylation and the expression of some miRNAs associated with metabolic diseases in the offspring through life.

## 5. Concluding Remarks

Several studies have indicated that children exposed to a GDM environment present higher susceptibility to the development of diseases later in life. In addition to genetic inheritance, epigenetic effects are essential to define individual wellness. GDM is a pregnancy condition in which epigenetic alterations occurring in the placenta may affect the fetus. Whether an epigenetic flow exists through the placenta from the mother to the fetus and vice versa is not clear and requires further research. Investigations into epigenetic alterations in the placenta due to GDM have been limited, and little is known about the epigenetic marks inherited by the offspring after in utero exposure to GDM conditions. Research limitations include ethical concerns regarding studying a newborn, complications related to long-term follow-up studies in humans, several variables associated with each participant, and the differential biology of the placenta between species when relying on animal models. For these reasons, umbilical cord blood, placental samples, and cell lines are most used to study the effects of exposure to GDM. These approaches also carry some limitations such as epigenetic changes that may be due to alterations in placental or cellular morphology or culture conditions but not due to GDM itself. In addition, genetic predisposition may influence the epigenetic signature; thus, it would be interesting to investigate cases where diabetic women are pregnant by surrogacy, as well as the epigenetic alterations in placentas from twins, to investigate individual changes occurring in the same environment. These and other investigations will help identify those epigenetic modifications in the placenta that are genuinely induced by GDM. 

Sometimes, the diagnosis of GDM is underestimated because the incidence may vary according to the ethnicity and to the diagnostic criteria used to detect it; thus, GDM is not always properly diagnosed. Understanding the epigenetic mechanisms and alterations caused by GDM in the placenta could help to understand and predict the susceptibility to disease after birth and further in life. 

Health organizations should inform couples planning a family about the effects of their habits on their offspring, as well as the importance of an accurate follow-up of pregnancy until delivery. Quality lifestyle, healthy food, and physical exercise are essential to avoid GDM, and they can help reduce the development of chronic diseases in future generations.

## Data Availability

No new data were created or analyzed in this study. Data sharing is not applicable to this article.

## Supplementary Materials

The following are available online at https://www.mdpi.com/article/10.3390/epigenomes5020013/s1, Table S1: Placental miRNA expression/Placental DNA methylation.
